# Effect of Low-Temperature Plasma Surface Treatment on Bonding Properties of Single-Lap Joint of Thermosetting Composites

**DOI:** 10.3390/polym15071631

**Published:** 2023-03-24

**Authors:** Liwei Wen, Xinying Xu, Lihua Qin

**Affiliations:** 1 College of Material Science and Technology, Nanjing University of Aeronautics and Astronautics, Nanjing 210016, China; 2 Department of Mechanical design, Beijing Aerospace Long March Vehicle Research Institute, Beijing 100048, China

**Keywords:** carbon fiber-reinforced epoxy resin composites, plasma surface treatment, single-lap joint, adhesive properties, surface physicochemical properties

## Abstract

Bonding is one of the main forms of composite bonding. In order to investigate the effect of low-temperature plasma surface treatment on the bonding properties of carbon fiber-reinforced epoxy resin composites (CF/EP), a single-lap joint of CF/EP was prepared. The surface of the CF/EP was treated with atmospheric pressure “low-temperature plasma spray” equipment, and the tensile shear strength, surface morphology, surface contact angle and surface chemical composition of the CF/EP before and after plasma treatment were characterized. Finally, the samples were treated with traditional sandblasting, compared and analyzed. The results show that the effect of low-temperature plasma surface treatment on CF/EP joints is better than that of traditional sandblasting treatment. After low-temperature plasma surface treatment, the tensile shear strength of the CF/EP single-lap joint increased by 119.59% at most, and the failure form of the joint changed from untreated interface failure to mixed failure dominated by cohesion failure. Plasma can etch the surface of composite materials, the mechanical interlock between the carbon fiber and glue is enhanced and the bonding performance of the composite is improved. In addition, after low-temperature plasma surface treatment, the introduction of a large number of oxygen-containing active groups such as C-O and C=O can increase the surface free energy, reduce the contact angle and improve the surface activity and wettability of the composites. However, too long a treatment time will lead to excessive plasma etching of carbon fibers, thus weakening the active effect of the oxygen-containing active groups on the surface of the composites, and the surface wettability is no longer improved, but the adhesive properties of CF/EP are reduced. This paper plays a guiding role in the bonding technology of composite materials.

## 1. Introduction

Carbon fiber-reinforced resin matrix composite (CFRP) has excellent properties such as high specific strength, light weight, fatigue resistance and corrosion resistance [1,2]. It has gradually become the preferred material in aerospace, automobile, construction and other fields [3], and has been widely used. At present, many complex carbon fiber composite structures on the market have to rely on specific connections to solve the problem of load transfer. However, in the whole structure, it is easy for the stress to concentrate in the joint, so this part is often weak and it is necessary to focus on ensuring the joint strength of the composite. Compared with other connection methods (such as traditional mechanical connection [4], welding [5], riveting, etc.), bonding has the advantages of high shear strength, light structure, uniform stress distribution, no galvanic corrosion and so on [6]. In addition, the cost advantage is very significant [7], so bonding technology has become a mature and popular method in composite bonding technology.

In order to improve the compressive strength after impact (CAI), some aeronautical composites such as M21C and X850 were toughened with epoxy resin with thermoplastic components to enhance the impact resistance. However, the addition of thermoplastic components affects the viscosity and process properties of the resin matrix, resulting in difficulties in bonding the interface between the resin and the adhesive [8,9]. In addition to the performance of the adhesive itself affecting the bonding performance of the composite adhesive structure, the surface properties of the substrate will also affect the bonding performance of the composite adhesive structure [10]. Surface pretreatment before bonding can prevent or eliminate weak boundary layers, which, if not eliminated in time, will hinder the close contact between the binder and the bonded atoms or molecules. This leads to stress relaxation and even fracture of the interface after bonding [11]. Therefore, the bonding properties of the composites can be improved with surface treatment.

Before bonding, the commonly used surface treatment methods include solvent cleaning, mechanical surface treatment, chemical surface treatment, plasma treatment, laser treatment and so on [12,13]. Among them, mechanical surface treatment (such as sandpaper grinding, traditional sandblasting, etc.) easily causes great damage to the substrate surface, and easily produces pollutants [14], chemical surface treatment (such as coupling agent coating, anodizing, acid etching, etc.) easily produces chemical emissions and other environmental problems and has low efficiency, and laser treatment has the risk of fiber damage and delamination [15]. Compared with other surface treatment methods, plasma surface modification has the advantages of a simple process, cleanness and environmental protection, time-saving and high efficiency [16], and its influence depth is only hundreds of nanometers; it only affects the surface of the fiber without changing the internal properties of the matrix [17]. It has become a new and effective method of interfacial modification, which is of great significance to improve the bonding properties of carbon fiber composite adhesive joints.

Some scholars at home and abroad have studied the application of plasma surface treatment technology in composite materials, metal materials and other fields. The results indicate that after plasma surface treatment, the adhesive strength and wettability of composite or metallic materials are greatly enhanced. Liu Zhe et al. [18] used air plasma to modify the surface of carbon fiber under the working conditions of 30 Pa and 200 W. It was found that the surface of the carbon fiber modified with plasma became rougher, the polarity was enhanced, and the adhesion between the carbon fiber and BMI resin was accordingly increased, and the interlaminar shear strength of the composites was significantly improved. Li Changqing et al. [19] used low-temperature air plasma to treat the surface of carbon fiber/epoxy resin composites. The results showed that the surface roughness of the material was improved, and the water contact angle decreased from 78° to 35.5°. The surface energy increased by about 2.3 times, and the oxygen content clearly increased after plasma treatment. Lin Jianping et al. [20] carried out atmospheric pressure jet plasma treatment on the surface of CFRP with three kinds of gases (argon, nitrogen and air). The results showed that when the plasma gas was nitrogen and air, the surface free energy of the CFRP substrate increased, polar chemical functional groups such as -NH2, -OH and -COOH were produced on the surface and the interfacial bonding strength between the CFRP and adhesive was improved. Encinas et al. [21] used APPT to treat the surfaces of two kinds of glass fiber-reinforced polymer matrix composites. The results showed that due to the action of plasma surface activation and surface etching, the bonding properties of the joints of the two materials were improved, and the failure mode of the joints also changed. Williams et al. [22] treated the surface of stainless steel and epoxy composites with atmospheric helium oxygen plasma. Through experiments, the lap shear strength of the stainless steel and epoxy composite samples increased by 80% and 150%, respectively. Therefore, it was concluded that the bonding force between stainless steel and epoxy composites is enhanced after plasma treatment. Comyn et al. [23] found that after plasma treatment, PEEK can form rich oxygen-containing groups such as -CO- and -OH on the surface of the material, which greatly increases the surface energy and improves the surface activity of the material. Chris et al. [24] used APT to treat a CFRP surface. They found that under the action of plasma, the surface contaminants were removed, the oxygen-containing chemicals on the surface increased and the surface wettability of the CFRP was improved.

In recent years, plasma surface modification technology has been gradually used in the bonding surface treatment of aerospace materials. However, in the past, many plasma surface treatments of composite materials were generally carried out under vacuum conditions [25], which needed to be completed under lower pressure. Vacuum discharge plasma equipment has the advantages of large plasma energy and uniform plasma distribution. However, the disadvantage is that it is necessary to build a vacuum cavity, the sample size is limited, it is not suitable for the treatment of continuous samples, the equipment is expensive and the cost is high [26]. In addition, most of the research on plasma surface modification technology in the field of composites suggests first treating the fiber with plasma, and then solidifying the treated fiber and resin matrix. However, there are few studies on the plasma surface treatment of the molded composites. Therefore, in this paper, non-vacuum “low-temperature plasma spray” equipment is used to treat the surface of carbon fiber epoxy resin composites under atmospheric pressure, and the temperature of the material surface is about 80 °C. The ablation on the surface of the material is very small. In this paper, a single-lap joint of carbon fiber epoxy resin composite (CF/EP) was fabricated, and the surface of the joint was treated with low-temperature plasma treatment and traditional sandblasting treatment, respectively, and then bonded. The surface morphology, wettability and chemical composition of the composite were characterized through tensile shear strength testing, scanning electron microscope (SEM) analysis, contact angle testing and X-ray spectroscopy (XPS) analysis. Combined with the bonding interface theory, the effect mechanism of low-temperature plasma surface treatment on the bonding properties and surface physicochemical properties of thermosetting composites was studied.

## 2. Experiment

### 2.1. Experimental Materials

In this experiment, the EH918 series prepreg produced by Hengshen Company is selected. The curing temperature is 180 °C, and the standard fiber area density of the unidirectional prepreg is 75–300 g/m^2^. The performance parameters of the EH918 resin and HF40C fiber are shown in Table 1 and Table 2, respectively. In addition, several fiberglass boards (custom size 82 mm × 150 mm × 2 mm), J-250 two-component adhesive provided by Heilongjiang Petrochemical Research Institute, 800-mesh fine sand paper and 150-mesh diamond powder 500 g are prepared.

### 2.2. Sample Preparation and Treatment Method

The single-layer thickness of the prepreg is 0.187 mm, 12 layers are laid unidirectionally through the 0 degree paving method and the carbon fiber epoxy resin composite plate is obtained using a vacuum at room temperature and curing in an oven at 180 °C. The thickness is about 2.24 mm. The composite laminate is cut into 14 pieces with a size of 100 mm × 150 mm × 2.24 mm using a water cutting machine.

**Figure 1:**
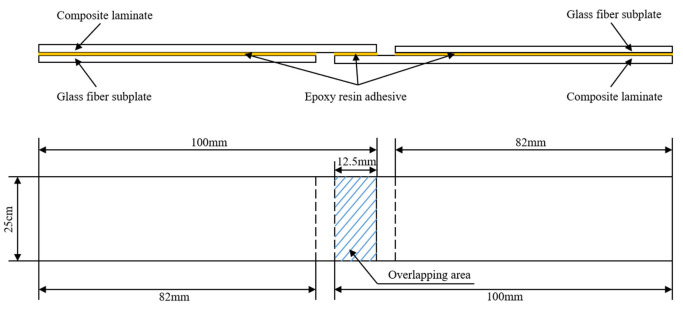
Schematic diagram of bonding process and final sample size.

The samples are divided into seven groups with two composite plates (100 mm × 150 mm × 2.24 mm) and fiberglass boards (82 mm × 150 mm × 2 mm) in each group. According to the mass ratio, J-250 adhesive resin (A): J-250 adhesive curing agent (B) = 1:1 is taken, mixed well and set aside. According to the test method of single-lap tensile shear strength of GB/T33334-2016 adhesive (composite to composite), the mixed adhesive film is uniformly applied on the surface of the composite plate and the bonding surface length is 12.5 mm. The glass fiber cushion plate is bonded to the sample with 82 mm, contact pressure 0.05 MPa, room temperature curing for 48 h. After the curing is complete, the lapped sample is cut to a standard size (that is, the base material size is 25 mm × 100 mm × 2.24 mm) with five pieces in each group. The schematic diagram of the bonding process and the final sample size diagram are shown in Figure 1.

Seven groups of samples need to undergo different surface treatments before bonding. The first group of samples is the blank group. Only 800-mesh fine sandpaper is used to polish the bonding area of the sample. After grinding, the surface of the sample is wiped with anhydrous ethanol and dried at room temperature. The second group of samples are sandblasted with a 220 L vertical box sandblasting machine, and the sandblasting particles are carborundum. The process parameters of the sandblasting are shown in Table 3.

**Figure 2:**
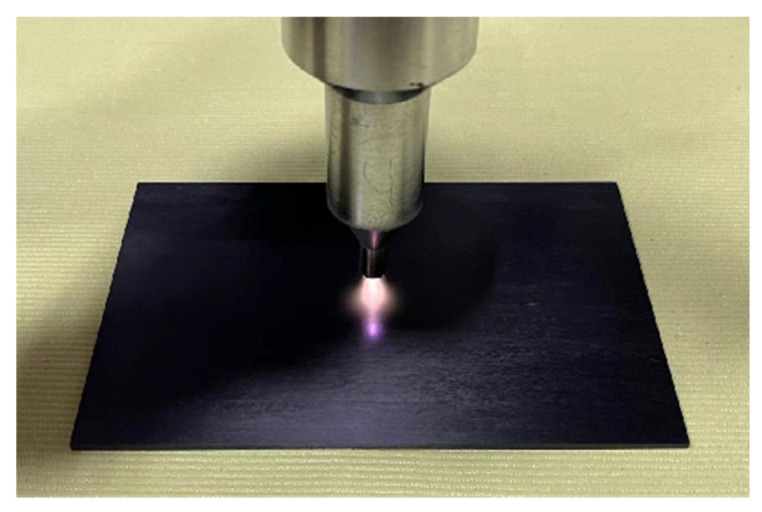
Surface of composite laminates treated with plasma.

**Figure 3:**
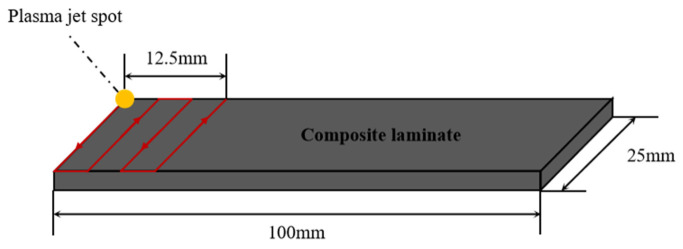
Schematic diagram of the motion path of plasma jet on composite laminates.

**Figure 4:**
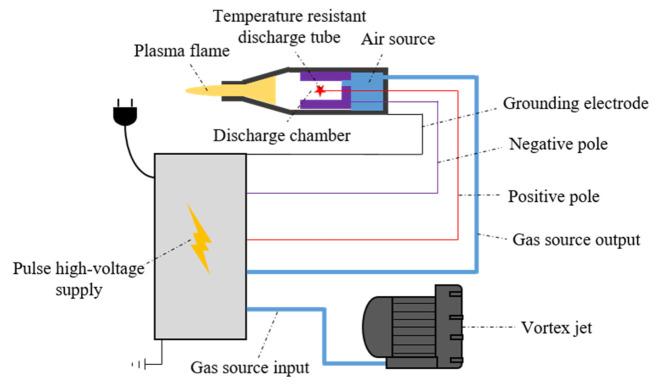
Schematic diagram of the working principle of “low-temperature plasma injection” equipment.

**Figure 5:**
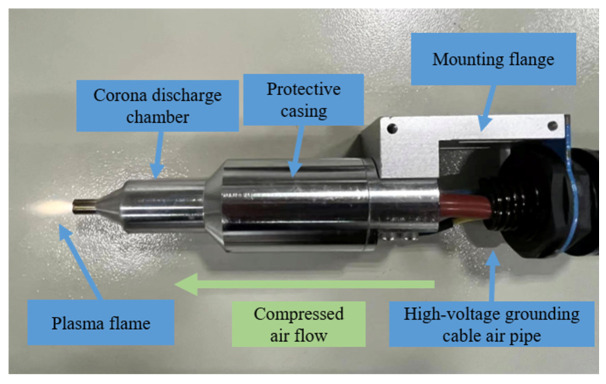
Physical diagram of low-temperature plasma spray gun.

The third to the sixth group of samples are still polished with fine sandpaper, cleaned and dried with anhydrous ethanol, and the power, injection speed and pressure of the plasma treatment equipment are adjusted to control the plasma treatment time of 25 s, 50 s, 125 s and 250 s, respectively. The distance from the nozzle to the sample is about 5 mm. The motion paths of the plasma-treated composite laminates and plasma jets on the composite laminates are shown in Figure 2 and Figure 3, respectively. The surface treatment of the seventh group of samples is 125 s after sandblasting. The “low-temperature plasma spray” equipment operates under atmospheric pressure, and the surface temperature of the composite is about 80 °C during treatment. The equipment consists of a plasma generator, a low-temperature plasma jet gun and a Haley vortex jet. The working principle diagram of the “low-temperature plasma spray” equipment and the physical diagram of the low-temperature plasma spray gun are shown in Figure 4 and Figure 5.

The main operating parameters of the low-temperature plasma surface treatment are discharge power, gas type, high-pressure gas flow rate, nozzle shape and diameter, as shown in Table 4.

### 2.3. Mechanical Property Test

**Figure 6:**
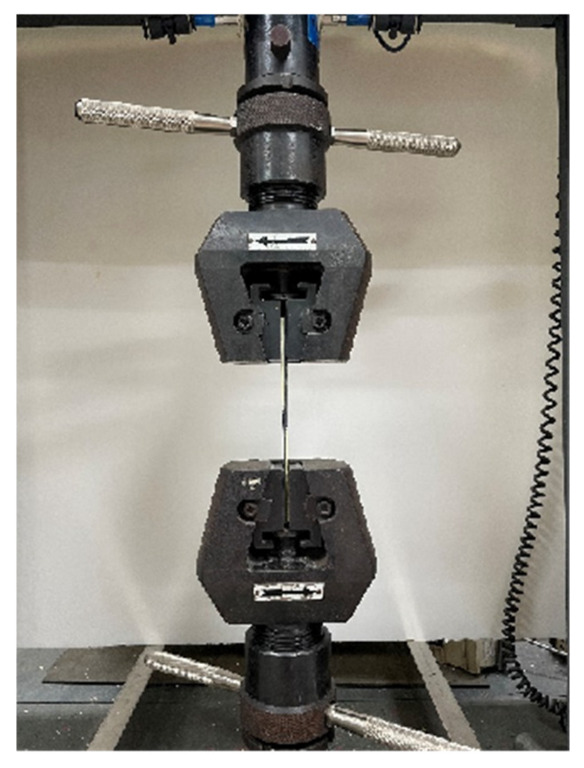
Picture of electronic universal testing machine.

The tensile and shear properties of a single-lap joint are tested with an electronic universal testing machine according to the GB/T 33334-2016 standard. The schematic diagram of the tensile shear test is shown in Figure 6.

The testing machine carries out the test at a constant loading rate. Under the axial load parallel to the lap surface, the specimen is brought to breaking point, and the maximum failure load is recorded. The average value of each group of samples is calculated based on five values, and the tensile shear strength of the CF/EP specimen at the lap joint is calculated from Formula (1).

(1) $$\tau =F_{m}/(B\times L)$$

where *τ* is the tensile shear strength (MPa), *F*_m_ is the ultimate failure load (N) and B and *L* are lap width and lap length, respectively, in mm. The macroscopic fracture morphology of the adhesive-bonded joint is observed, the fracture form of the joint is analyzed and the failure type of the joint is judged.

### 2.4. Surface Topography Observation

An Oxford Quorum SC7620 sputtering coating instrument is used to spray gold on the surface of the CF/EP single-lap joint for 45 s and 10 mA, and then the fracture morphology of the adhesive joint is photographed with a ZEISS Sigma 300 scanning electron microscope. The acceleration voltage is 3 kV.

### 2.5. Contact Angle Test

**Figure 7:**
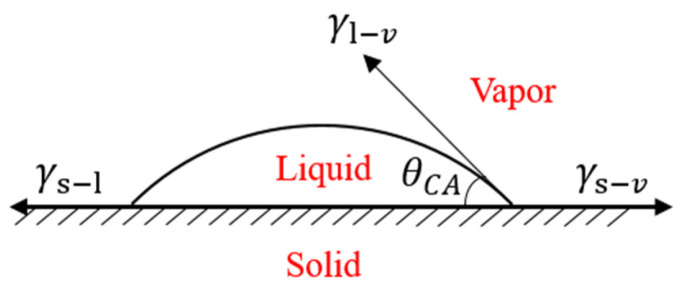
Contact angle diagram.

The CF/EP laminates are cut into 30 mm × 20 mm × 2.24 mm samples, and the surfaces are treated with no treatment, sandblasting, plasma treatment for 60 s, plasma treatment for 120 s, plasma treatment for 300 s, plasma treatment for 600 s and plasma treatment for 120 s after sandblasting. The reason for this arrangement of the experiment is that the duration of plasma treatment is almost positively related to the area of surface treatment on the sample. The contact angles of the CF/EP surfaces with different surface treatments are measured with a JC2000D2S contact angle measuring instrument. The contact angles at different positions on the surface of each group of samples are measured 5 times, and the average values of the measured data are taken. The droplet volume is 2 μL, and the selected test liquid is deionized water and ethylene glycol. The contact angle diagram is shown in Figure 7. θ_CA_ is the contact angle of the droplet on the sample surface, and its size is the angle between the solid–liquid interface and the gas–liquid interface.

As shown in Table 5, the surface energy parameters, polar components and dispersion components of the two test liquids are known. Combined with the contact angle and interfacial tension theory of the two test liquids, the surface free energy of the composites is calculated according to Owens–Wendt–Kaelble [27] method.

(2) $$\gamma_{L}(1+\cos\theta )=2\sqrt{\gamma_{S}^{d}\gamma_{L}^{d}}+2\sqrt{\gamma_{S}^{p}\gamma_{L}^{p}}$$

(3) $$\gamma_{S}=\gamma_{S}^{d}+\gamma_{S}^{p}$$

In Formulas (2) and (3), $\gamma_{L}\;$ is the surface energy of the test liquid, θ is the contact angle, $\;\gamma_{S}^{d}$ and $\gamma_{S}^{p}$ represent the dispersion component and polar component of the surface energy of the composite sample, respectively, $\gamma_{L}^{d}$ and $\gamma_{L}^{p}$ represent the dispersion component and polar component of the test liquid surface energy, respectively, and $\gamma_{S}$ is the total surface free energy of the composite sample.

### 2.6. X-ray Spectroscopy (XPS) Analysis

In order to further study the effect of low-temperature plasma surface treatment on the physical and chemical properties of the composite surface (such as the changes in surface element content and group), the element content and functional group content of the composite surface are measured using an X-ray photoelectron spectrometer, the XPS scanning energy spectrum is processed using the least square fitting software Avantage and the C 1 s, N 1 s and O 1 s fine spectra are fitted with peak separation. The types and contents of chemical elements and functional groups on the surface of the CF/EP samples treated with different surface treatments are quantitatively analyzed. The CF/EP laminates are cut into 5 mm × 5 mm × 2.24 mm samples, and the surfaces are treated with no treatment, sandblasting, plasma treatment for 5 s, plasma treatment for 10 s, plasma treatment for 25 s, plasma treatment for 60 s and plasma treatment for 25 s after sandblasting. The reason for this arrangement of the experiment is that the duration of plasma treatment is almost positively related to the area of surface treatment on the sample. The experiment is analyzed using an American Thermo Science K-AlphaX photoelectron spectrometer. The X-ray photoelectron spectrometer uses AlKα rays as the excitation source (hv = 1486.6 eV). The full-spectrum scanning step is 1 eV and the narrow-band step is 0.1 eV.

## 3. Results and Analysis

### 3.1. Effect of Low-Temperature Plasma Surface Treatment on Mechanical Properties of Composites

In order to explore the effect of plasma treatment on the bonding strength of composites, specimens of single-lap joints were made, and tensile shear tests were carried out by using an electronic universal testing machine. By changing the time of plasma treatment, the tensile shear strength of the composite single-lap joint shows significant changes compared with the untreated sample and the sandblasted sample. Table 6 shows the tensile shear strength of the CF/EP single-lap joints with different surface treatments.

As can be seen from the results in the table, the tensile shear strength of the untreated CF/EP single-lap joint is only 9.65 MPa, and the tensile shear strength increases by 31.4% after sandblasting. The tensile shear strength increases by 72.85%, 119.59% and 63.52%, respectively, after plasma treatment for 25 s, 50 s and 125 s. When the time of plasma treatment is extended to 250 s, the tensile shear strength decreases to 11.85 MPa, which is slightly lower than that after sandblasting, and only 22.8% higher than that of the original sample, and the treatment effect is poor. The tensile shear strength of the sandblasted and plasma-treated samples is lower than that of the untreated samples for the following reasons. The combination of sandblasting and plasma treatment has too strong an etching effect on the surface of the composites, which destroys the carbon fiber substrate, resulting in the epoxy resin adhesive being unable to combine well with the carbon fiber.

**Figure 8:**
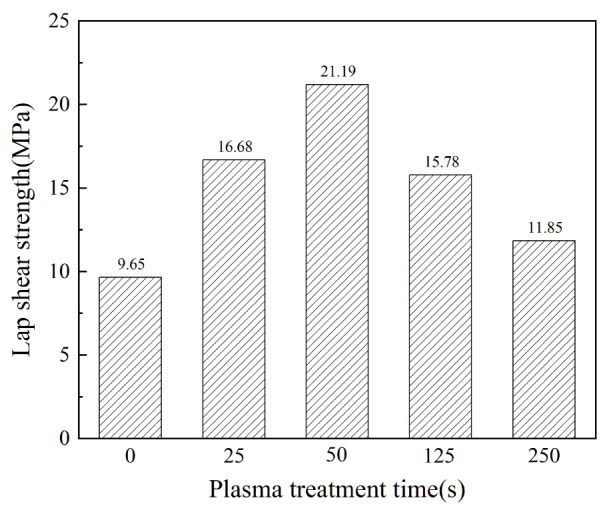
Tensile shear strength of CF/EP single-lap joint after different plasma treatment times.

Figure 8 shows the changing trend of the tensile shear strength of the composite single-lap joints after different plasma treatment times.

Through analysis, it is concluded that with the extension of the plasma treatment time, the tensile shear strength of the CF/EP single-lap joint increases at first and then decreases. When the plasma treatment time is 50 s, the tensile shear strength of the CF/EP single-lap joint reaches its peak, and the bonding property of the composite is the best. When the treatment time is extended to 125 s or even 250 s, the tensile shear strength does not increase further, but has a downward trend, which may be due to the over-etching of plasma and the decrease in oxygen-containing groups.

These results show that plasma treatment plays a clear role in improving the bonding properties of composites, but it is not the case that the longer the treatment time is, the better the treatment effect is. Too short or too long a treatment time will lead to no clear improvement in the bonding properties of the composites. First of all, with the increase in treatment time, the concentration of active particles in the plasma increases correspondingly. The higher the etching probability of these active particles and carbon fiber surface reaction, the higher the surface roughness of the composites. Furthermore, the effective contact area between the carbon fiber and epoxy resin adhesive increases, the mechanical bonding strength increases, the mechanical properties of the composites are improved and the interfacial bonding properties are better. However, when the treatment time is too long, gradually etching the exposed carbon fiber in the extended treatment time will seriously damage the carbon fiber network, deteriorate the mechanical properties of the composites after processing and finally lead to the decrease in tensile shear strength.

In addition to the tensile shear strength of the single-lap joint of the composite, the fracture form of the joint is also an important index to measure the bonding properties of the composite. The failure types of joints [28,29] are usually interface failure, cohesion failure, substrate failure and mixed failure (a combination of multiple failure forms), as shown in Table 7.

Table 8 shows the macroscopic fracture morphology and failure forms of composite single-lap joints with different surface treatment methods after tensile shear strength testing.

By analyzing the fracture morphology of the joint with the various surface treatment methods in the table, it can be found that the fracture surface of the composite joint without treatment and sandblasting treatment is more flat and smooth, and the epoxy resin adhesive is almost uniformly distributed on one side of the bonding face. The failure form of the joint is interface failure (adhesive–substrate interface failure). The results show that after no treatment and sandblasting treatment, the interfacial bonding between the composite and the adhesive is poor, and the interfacial bonding strength of the composite is relatively low, which makes it is easy to form a weak bonding interface and causes the joint to fracture prematurely on the weak bonding interface. After plasma treatment for 25 s, 50 s and 125 s, the failure mode of the CF/EP joint is mixed failure, which is the combination of interfacial failure and cohesion failure (internal failure of adhesive layer). Part of the failure occurs in the interior of the adhesive, and the bonding strength of the composite is higher than that of the untreated and sandblasted samples. After plasma treatment for 250 s, the failure mode of the joint is the combination of carbon fiber substrate failure and interface failure. The failure form of the joint treated with plasma after sandblasting is the mixed failure, the combination of substrate failure and cohesion failure.

The above results show that plasma treatment can improve the interfacial bonding strength of the composites and change the failure form of the single-lap joints. With the increase in plasma treatment time, the failure form of the single-lap joint gradually changed from interfacial debonding failure between the adhesive and CF/EP to mixed failure dominated by cohesion failure (the combination of cohesion failure and interfacial failure), and then to cohesion failure, which mainly occurred in the interior of the adhesive layer, indicating that the bonding strength of most areas of the adhesive interface was higher than that of the adhesive, and the properties of the adhesive were fully utilized. The interfacial bonding strength of the corresponding composites increased at first and then decreased. When the plasma treatment time continued to extend, the failure form of the joint was a mixed failure dominated by substrate failure (the combination of substrate failure and interface failure), and the interfacial bonding strength of the composites was further reduced. This shows that excessive plasma treatment cannot improve the bonding properties of the composites.

### 3.2. Effect of Low-Temperature Plasma Surface Treatment on Surface Morphology of Composites

**Figure 9:**
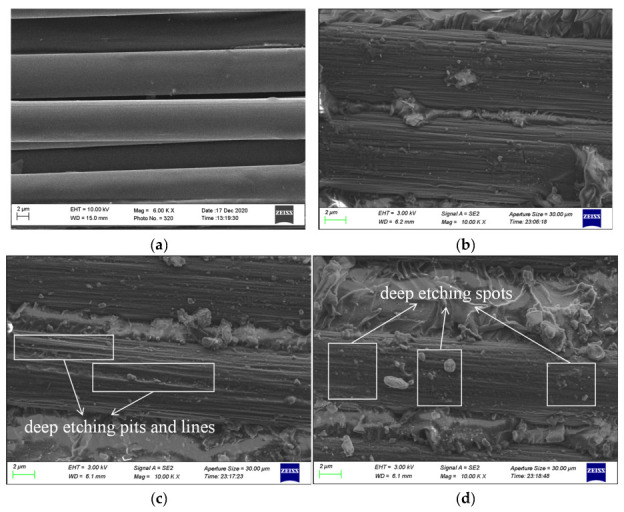
Surface morphology of CF/EP single-lap joints with different surface treatments. (**a**) Untreated, (**b**) sandblasting treatment, (**c**) plasma treatment for 50 s and (**d**) plasma treatment for 125 s.

In order to study the effect of plasma treatment on the surface morphology of composite single-lap joints, the surface morphologies of composite single-lap joints after different treatment methods were characterized using a scanning electron microscope (SEM). The overall macroscopic morphology of the carbon fiber surface and the etching process caused by the plasma can be seen well in the high-resolution scanning electron microscope images. Figure 9a–d shows the surface morphology of the CF/EP single-lap joints with different surface treatment methods.

The surface morphology of the untreated CF/EP single-lap joint is shown in Figure 9a. It can be clearly seen from the figure that the exposed carbon fiber surface of the original untreated composite surface does not adhere to more resin, the fiber surface is clean and smooth and the fiber surface has almost no groove texture distributed along the axis. Figure 9b shows the surface of the carbon fiber after sandblasting, and a shallow longitudinal strip texture can be observed on the surface of the carbon fiber, indicating that emery blasting has a slight etching effect on the fiber, but this is not obvious.

Figure 9c,d shows the morphology of the carbon fiber on the surface of the composite after plasma treatment for 50 s and 125 s, respectively. The surface of the carbon fiber adheres to more resin and binds more closely with the epoxy resin glue. The surface of the carbon fiber becomes very rough, the longitudinal texture becomes deeper and shows a gully shape and there are some etching pits, lines and spots on the surface, with clear plasma etching marks. The bombardment of the carbon fiber surface by high-energy particles or free radicals in the plasma is an important reason for these changes on the surface of the carbon fiber after plasma treatment. Due to the influence of etching and cleaning after plasma modification, the outermost layer of the fiber surface is removed, resulting in the reduction in weak boundary conditions that may exist in the composites, thus improving the adhesive properties of the composites. In addition, because the surface profile of the carbon fiber is rough, there are many gullies and protrusions, and there are pores conducive to adhesive penetration in some areas, the bonding area between the carbon fiber and adhesive is greatly increased and the plasma etching effect is obvious. The glue can be connected not only with the resin matrix but also with the fiber at the same time, and the etched rough surface improves the mechanical interlock strength. As a result, the epoxy resin adhesive can effectively adhere to the surface of the carbon fiber, which has a favorable effect on the bonding properties.

When the treatment time is extended to 125 s, the grooves and spots on the carbon fiber surface gradually widen, deepen and increase, more granular and linear protruding structures appear on the surface and the plasma etching marks become more obvious. Plasma sputtering may damage the atomic chain on the surface of the carbon fiber, break it and shoot down some atoms, and low molecules spray out the surface of the carbon fiber and leave voids. However, these atoms and atomic chains may be polymerized into relief with the increase in plasma processing time. This is the reason why the surface of the carbon fiber becomes rougher with the extension of plasma treatment time, and it also confirms that when the treatment time increases from 50 s to 125 s, the tensile shear strength of the composite single-lap joint decreases from 21.19 MPa to 15.78 MPa.

**Figure 10:**
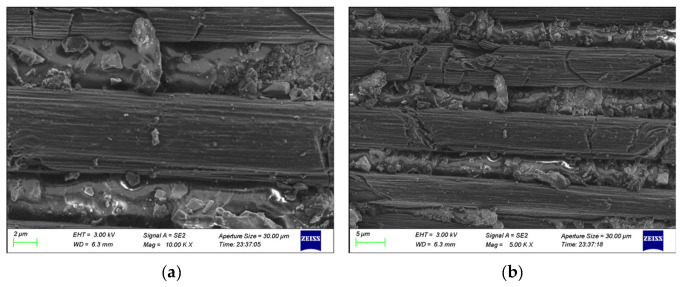
Surface morphology of CF/EP single-lap joints with sandblasting and plasma treatment for 125 s at (**a**)10 k times magnification and (**b**) 5 k times magnification.

Figure 10a,b shows the surface morphology of the single-lap joint treated with sandblasting followed by plasma treatment for 125 s. Figure 10a,b shows the morphology of the carbon fiber on the composite surface after 10 k and 5 k times magnification with a scanning electron microscope, respectively. As shown in the figure, the carbon fiber surface still has structural characteristics such as gully grooves and raised spots, but in addition, radial cracks are also observed. These cracks are deep or shallow and unevenly distributed on the surface and interior of the carbon fiber, indicating that the combination of sandblasting and plasma treatment leads to a certain degree of damage to the fiber. The phenomenon of resin thermal damage and local melting appeared on the surface of the composite, more resin adhered to the carbon fiber, the carbon fiber was embedded in the resin, the bonding energy of the CF/EP surface resin–carbon fiber interface became weaker, and the surface resin fell off easily with the peeling off of the adhesive, thus reducing the bonding properties of the composites.

From the above analysis, it can be concluded that the surface morphology of the CF/EP single-lap joint changes clearly before and after plasma treatment. With the extension of plasma treatment time, the etching effect of the plasma on the composite surface is gradually enhanced, and the morphology of the carbon fiber on the composite surface changes from a smooth surface to a rough surface with many grooves and protuberances. The bonding properties of the composites continue to improve, but when the treatment time is too long, the carbon fiber undergoes some damage due to the excessive plasma etching, which affects the bonding properties of the composites.

### 3.3. Effect of Low-Temperature Plasma Surface Treatment on Surface Wettability of Composites

**Figure 11:**
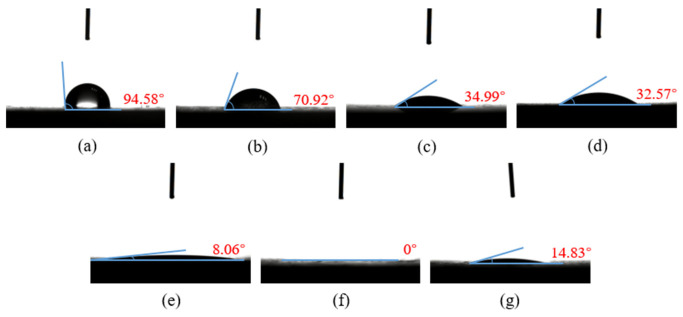
Contact angle between CF/EP surface and deionized water with different surface treatments. (**a**) Untreated, (**b**) sandblasting treatment, (**c**) plasma treatment for 60 s, (**d**) plasma treatment for 120 s, (**e**) plasma treatment for 300 s, (**f**) plasma treatment for 600 s and (**g**) sandblasting and plasma treatment for 120 s.

The surface wettability of composites can be characterized using surface contact angle and surface free energy. In this paper, the contact angles of the composites with different surface treatments for deionized water and ethylene glycol were measured using a contact angle measuring instrument. Figure 11a–g is the contact angle between the surface of the CF/EP sample and deionized water.

It can be seen from Figure 11 that the contact angle between the untreated CF/EP surface and deionized water is the largest at 94.58°. At this time, the CF/EP surface is hydrophobic and has poor wettability, which is disadvantageous to the adhesion of the composites. After sandblasting, the contact angle between the CF/EP surface and deionized water is 23.66° lower than that without sandblasting treatment. After low-temperature plasma surface treatment, the contact angle between the composite surface and deionized water further decreased, the CF/EP surface changed to hydrophilic and the wettability became better. This is due to the fact that the high-energy particles in the plasma remove the surface pollutants and introduce polar free radicals into the surface to improve the surface wettability of the samples. With the increase in treatment time, the contact angle between the composite surface and deionized water decreases gradually, especially when the plasma treatment time is 600 s, at which point the contact angle is 0°, the water droplets have been completely infiltrated into the surface of the CF/EP sample, the original shape of the water droplets cannot be observed on the sample surface and the wettability of the composite surface is the best. The contact angle of the deionized water on the surface of the sample treated with sandblasting and plasma treatment is slightly larger than that of plasma treatment for 600 s, indicating that the combination of sandblasting and plasma treatment does not further improve the wettability of the composite surface.

**Figure 12:**
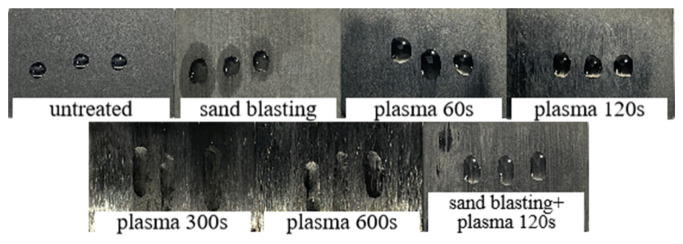
Morphological diagram of ethylene glycol droplets on the surface of CF/EP with different treatment methods.

Figure 12 shows the morphology of ethylene glycol droplets on the surface of the CF/EP with different surface treatments. It can be seen from the figure that after different surface treatments, the morphology of the droplets on the surface of the composite is different. The droplets on the untreated sample surface look full and show the shape of round droplets; after plasma treatment for 60 s and 120 s, the droplets on the sample surface tend to expand slightly, while after plasma treatment for 300 s and 600 s, the droplets on the sample surface spread clearly, mainly showing a spreading shape, but because the texture of ethylene glycol is sticky, it shows a strip shape on the sample surface. Overall, with the increase in plasma treatment time, the surface area of the droplets looking down from the top of the sample becomes larger and larger, and no longer increases when the treatment time is 600 s. The morphology of the droplets on the sample surface after sandblasting and plasma treatment for 120 s is similar to that of plasma treatment for 60 s, which shows that the combination of sandblasting and plasma treatment cannot improve the surface wettability of composites as well as plasma treatment in a short time.

**Figure 13:**
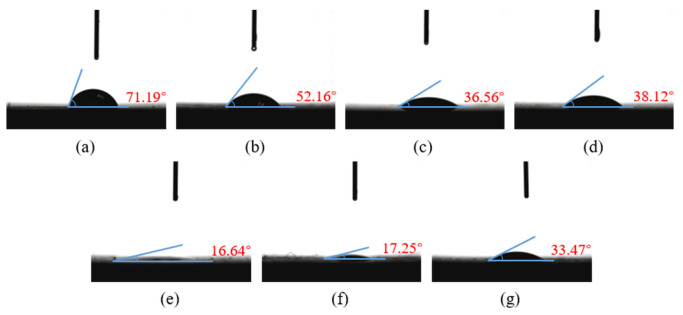
Contact angle between CF/EP surface and ethylene glycol with different surface treatments. (**a**) Untreated, (**b**) sandblasting treatment, (**c**) plasma treatment for 60 s, (**d**) plasma treatment for 120 s, (**e**) plasma treatment for 300 s, (**f**) plasma treatment for 600 s and (**g**) sandblasting and plasma treatment for 120 s.

Figure 13a–g is the contact angle between the surface of the CF/EP sample and ethylene glycol liquid. It is not difficult to see that the change trend of the ethylene glycol contact angle on the composite surface is essentially consistent with that of the deionized water contact angle on the composite surface.

The wettability of the composite surface is not only related to the surface contact angle, but also closely related to the surface free energy [30]. The increase in the surface energy of the composites can improve the wettability and provide favorable conditions for the bonding of the composites. In order to further evaluate the wettability of the composite surface, the total surface energy, polar component and dispersion component (non-polar component) of the CF/EP samples with different treatment methods were calculated using the Owens–Wendt–Kaelble formula, as shown in Table 9.

From the results in the table, it can be seen that the surface free energy of the untreated composite is lower, only reaching 23.18 mJ/m^2^, the dispersion component is larger than the polar component and the hydrophilicity of the composite surface is poor. After sandblasting, the surface energy of the composite is higher than that of the original material 9.39 mJ/m^2^, and the polar component of the surface energy of the composite exceeds the dispersion component. After plasma treatment, the surface energy of the composites is significantly improved, ranging from 50.12 mJ/m^2^ to 67.98 mJ/m^2^ compared with the untreated surface energy, the polar component of the surface energy of the composites is much larger than the dispersion component and the hydrophilicity of the composite surface is enhanced. The surface energy of the sample after sandblasting and plasma treatment is improved to 97.49 mJ/m^2^. Combined with the contact angle of this treatment in the previous paragraph, it can be concluded that the wettability of the composite surface with the combination of sandblasting and plasma treatment is not as good as that after plasma treatment.

**Figure 14:**
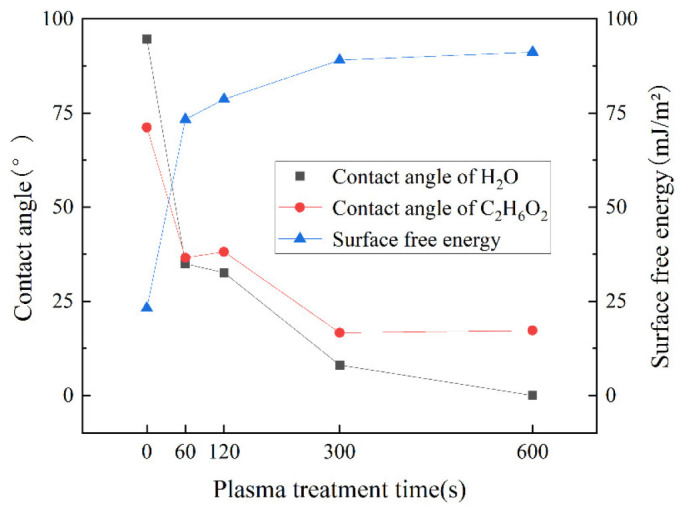
Effect of plasma treatment time on surface contact angle and surface free energy of CF/EP.

Figure 14 shows the relationship of the contact angle between the CF/EP surface and deionized water, the contact angle with ethylene glycol and the surface free energy of the composites with plasma treatment time.

The results show that without plasma treatment, the contact angle of the composite surface is very large, the surface free energy is low and the wettability is poor, but with the plasma treatment time of 60 s, the contact angle of the composite surface decreases substantially, the surface free energy also increases to a great extent and the wettability becomes better. When the time of the plasma treatment is extended from 300 s to 600 s, the contact angle and surface free energy of the composite surface have no clear change, but tend to be flat in the figure. This shows that the wettability of the composite surface can be significantly improved by plasma treatment for a short time, and when the plasma treatment time reaches a certain point, the treatment time will continue to increase but have almost no effect on the surface wettability of the composite. The wettability is no longer improved due to the extension of plasma treatment time.

**Figure 15:**
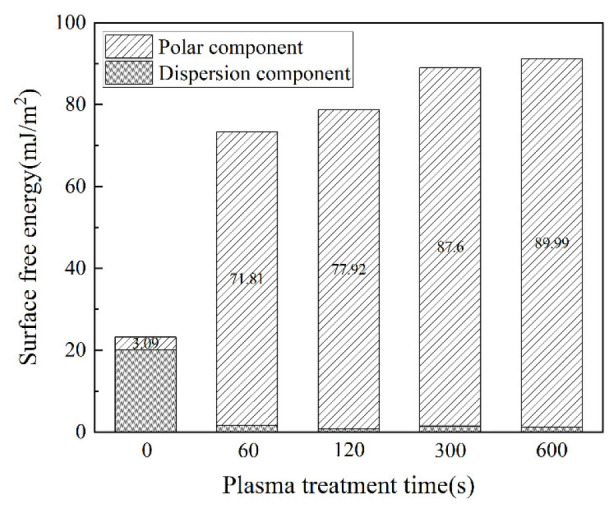
Surface free energy of CF/EP with different plasma treatment times.

Figure 15 is the surface free energy of the CF/EP composed of corresponding polarity and dispersion components at different plasma treatment times.

It can be seen from the figure that the composition of each component of the surface free energy of the composite changes from a high proportion of non-polar components without treatment to a high proportion of polar components after plasma treatment. After plasma treatment, the dispersion component of the surface energy of the composite has almost no clear change, while the polar component increases gradually with the extension of the plasma treatment time, which indicates that the plasma treatment is mainly due to the fracture of the molecular chain on the surface of the matrix caused by high-energy active particles and the grafting of more polar oxygen-containing groups onto the surface of the composite. Thus, the adsorption property between the adhesive and the surface of the composite is enhanced by increasing the content of the polar component in the surface free energy of the composite, and then the wettability of the surface of the composite is improved. When the plasma treatment time is 600 s, the total surface energy is only slightly higher than that when the plasma treatment time is 300 s. This shows that the longer treatment time cannot linearly improve the surface energy. In addition, according to the adhesive properties analyzed above, it can be found that with the decrease in the contact angle of the composite surface, the strength of the single-lap joint will not increase significantly. This is because there is a wetting envelope on the surface of the composite. The wetting and adsorption properties of the adhesive have a saturation value. There is no way to achieve the continuous improvement of the bonding properties of composite single-lap joints with the further improvement in surface wettability.

### 3.4. Effect of Low-Temperature Plasma Surface Treatment on Surface Chemical Composition of Composites

**Figure 16:**
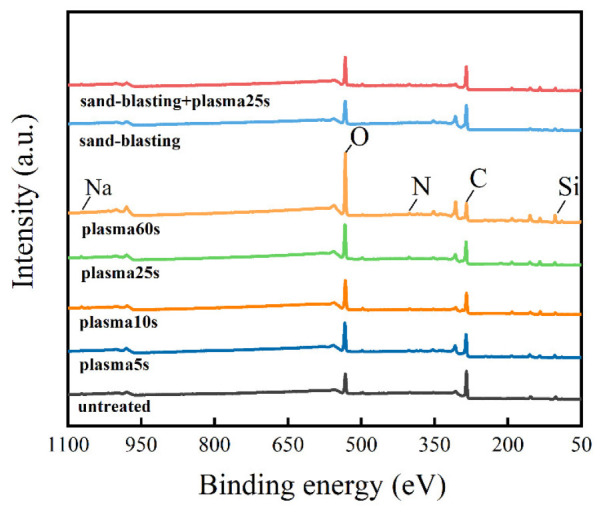
XPS full spectrum of CF/EP sample surface with different surface treatments.

Low-temperature plasma surface treatment can not only enhance the wettability of the composite surface by increasing the surface free energy of the composite, but also affect the chemical composition and content of the composite surface through the chemical reaction of the high-energy active particles in the plasma, so as to activate the surface of the composite. Therefore, in order to further study the effect of low-temperature plasma surface treatment on the physical and chemical properties of the composite surface (such as the changes in surface element content and group), the element content and functional group content of the composite surface were measured using an X-ray photoelectron spectrometer, the XPS scanning energy spectrum was processed using the least square fitting software Avantage and the C 1 s, N 1 s and O 1 s fine spectra were fitted with peak separation. The types and contents of chemical elements and functional groups on the surface of the CF/EP samples treated with different surface treatments were quantitatively analyzed. Table 10 shows the element composition and their corresponding contents on the surface of the CF/EP samples after different surface treatments (the percentage of element content shown in the table is the element content without removing the impurities such as P 2p, Mg 1 s, Ca 2p, etc.). The XPS full spectrum of the CF/EP sample surface with different surface treatment methods is shown in Figure 16.

Combined with the composition and content of different elements in Table 10 and the XPS full spectrum, it can be seen that the surface of the composite sample contains C, N, O, Si, Na and other elements, and their contents have changed clearly with different surface treatment methods. Among them, the content of C element and O element is the highest, because the testing range of XPS is the element of several nanometers on the surface of the material, so the carbon pollutants, resin and carbon fiber on the surface of the composite make the elements on the surface of the sample mainly C and O. The appearance of the silicon atomic peak is due to the residual components of the release agent on the surface of the composite. The figure also shows the sodium atom peak on the surface of the composite, and the trace amount of Na on the surface of the sample is likely to from contamination by accidental hand contact with the sample during the experiment.

As can be seen from the results in Table 10, the contents of C, N and O on the surface of the untreated CF/EP samples are 69.48%, 1.75% and 23.15%, respectively, and the contents of N and O are lower. After sandblasting, the content of C, N and O did not change clearly, but the content of C on the surface of the sample decreased linearly compared with the original sample, the content of N increased in varying degrees and the content of O increased steadily with the increase in plasma treatment time. The content of Si element on the surface of the untreated CF/EP sample is 7.07%, which decreases slightly after plasma treatment, because the plasma treatment process is effective in removing silicon residues in the carbon fiber. When the time of plasma treatment was gradually extended from 5 s to 60 s, the content of C element on the surface of the sample decreased to 32.09%. The proportion of O element rose to 44.34%, indicating that the carbon pollutants on the surface of the composite could be removed through plasma treatment, and the oxygen in the air was ionized and adsorbed to the surface of the composite. When the plasma treatment time was 25 s, the content of N element was 3.7%, which is more than twice the content of N element in the untreated sample, but after extending the time of plasma treatment, the content of N element was reduced by 1.31%. This shows that low-temperature plasma treatment can quickly graft a large number of nitrogen-containing active groups onto the surface of the composites. These nitrogen-containing groups come from nitrogen in the air, but because the content of oxygen-containing groups in the plasma is higher and their activity is stronger, they will replace the active nitrogen-containing groups on the surface of the CF/EP after a longer treatment time. Table 11 summarizes the oxygen–carbon ratio and nitrogen–carbon ratio of the CF/EP samples before and after plasma treatment.

It can be seen from Table 11 that the oxygen–carbon ratio and nitrogen–carbon ratio of the CF/EP surface before plasma treatment are lower, at 0.35 and 0.03, respectively. After plasma treatment, the ratio of oxygen to carbon increased greatly, and the ratio of oxygen to carbon increased linearly with the extension of treatment time. When the time of plasma treatment reached 60 s, the ratio of oxygen to carbon reached 1.38. The content of O element on the surface of the composite even exceeded that of C element. This shows that the existence of electrons and active oxygen atoms in the plasma makes the oxygen-containing active groups rapidly graft onto the surface of CF/EP, which increases the surface polarity of the composites, which is helpful to improve the bonding properties of the composites. Plasma treatment also had a certain effect on the nitrogen–carbon ratio, but the change was not significant. With the gradual extension of plasma treatment time from 5 s to 25 s, the nitrogen–carbon ratio of the CF/EP surface slightly increased, but when the plasma treatment time continued to extend to 60 s, the nitrogen–carbon ratio did not increase. The results show that the content of nitrogen-containing groups grafted onto the surface of the composites after plasma treatment is lower than that of oxygen-containing groups, and the oxygen-containing groups play an important role in improving the surface polarity of the composites.

**Figure 17:**
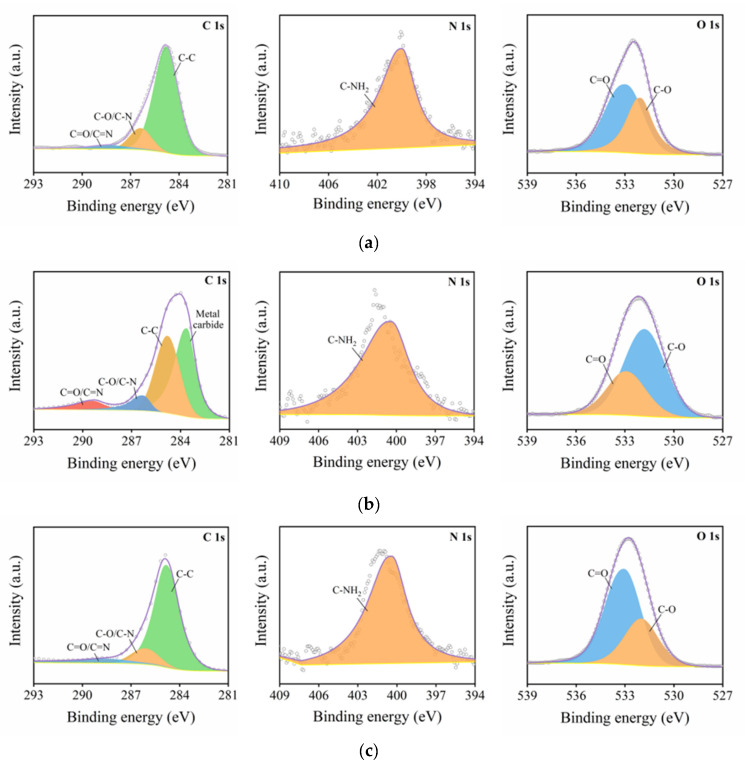

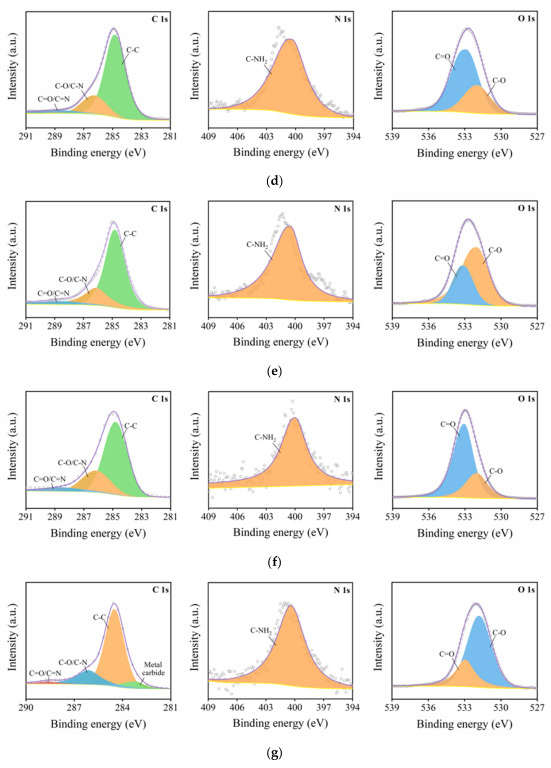
Peak separation fitting diagram of C 1 s, N 1 s and O 1 s on CF/EP sample surface before plasma treatment and with different plasma treatment times. (**a**) Untreated, (**b**) sandblasting treatment, (**c**) plasma treatment for 5 s, (**d**) plasma treatment for 10 s, (**e**) plasma treatment for 25 s, (**f**) plasma treatment for 60 s and (**g**) sandblasting and plasma treatment for 25 s.

Figure 17 shows the peak separation and fitting diagram of C 1 s, N 1 s and O 1 s on the surface of the CF/EP samples before and after plasma treatment. From the fitting results of the C 1 s fine spectrum peak separation, the types and corresponding contents of the surface functional groups of the CF/EP samples before and after plasma treatment can be obtained, as shown in Table 12.

For all the C 1 s, N 1 s and O 1 s fine spectra, the number of peaks and the types of functional groups in the spectrum did not change before and after plasma treatment. There are three main peaks in the C 1 s spectrum, and the corresponding functional groups are C-C, C-O (or C-N) and C=O (or C=N), and the corresponding binding energies are 284.8 eV, 286.11~286.38 eV and 288.44~288.58 eV, respectively. The functional group in the N 1 s spectrum is C-NH2 and its binding energy is 400~400.5 eV. There are two main peaks in the O 1 s spectrum, the corresponding functional groups are C-O and C=O and the corresponding binding energies are 532~533.07 eV and 532~533.06 eV. According to the area of the peak of each group in the C 1 s spectrum, the corresponding content can be calculated.

As can be seen from the results in Table 12, the proportion of each group on the surface of the composite changes with the time of plasma treatment. Without plasma treatment, the content of the C-C group on the surface of the composite was higher, while the content of the C-O and C=O groups was lower; after plasma treatment for 10 s, the content of the C-C group decreased from 78.7% to 71.4%, while the content of the C-O (or C-N) and C=O (or C=N) groups increased from 14.2% and 7.1% to 19.3% and 9.3%, respectively. This shows that the attack of the high-energy plasma on the C-C bond and graphitized carbon atoms is dominant, which activates it and interacts with the active groups in the plasma. Because the nitrogen content on the surface of the sample is low, most of the active groups on the surface of the composites are oxygen-containing active groups. This shows that a large number of high-energy active particles such as O+ and O· in the air plasma will react with the chemical groups on the surface of the composites to form polar oxygen-containing active groups with carbon–oxygen single bonds and carbon–oxygen double bonds, such as hydroxyl, carboxyl, carbonyl, etc. These active groups will be grafted onto the surface of the composites to improve the surface activity of the composites, which is conducive to the bonding between the carbon fiber and epoxy resin adhesive, thus improving the interfacial bonding properties of the composites. With the increase in plasma treatment time from 5 s to 25 s, the content of the C-C group on the surface of the composite decreased gradually, and the content of the C-O and C=O groups also showed a steady upward trend; when the plasma treatment time was extended from 25 s to 60 s, the content of the C-C group no longer decreased, and the C=O group decreased by 2%, but the total amount of oxygen-containing groups did not change compared with the treatment time of 25 s. This phenomenon shows that with the extension of plasma treatment time, the degree of hydroxyl-, carboxyl- or carbonylation of active particles in plasma further deepens in the process of contact with carbon fiber, thus improving the surface activity of the composites. However, too long a plasma treatment time will lead to serious etching on the surface of carbon fiber, resulting in a new interface layer on the surface of the composite, and some oxygen-containing groups will be stripped off, thus weakening the active effect of oxygen-containing groups on the surface of the composite.

Table 12 also shows the ratio of the content of polar groups (C-O, C=O) and non-polar groups (C-C) on the surface of the CF/EP samples with different plasma treatment times. The ratio of polar groups to non-polar groups on the surface of the sample without plasma treatment was 0.27. With the progress of plasma treatment, the ratio of polar groups to non-polar groups gradually increased, and when the treatment time was 25 s, the ratio of polar groups to non-polar groups was the highest, at 0.51, when the surface of the composite was grafted with the most oxygen-containing active groups. The treatment time continued to increase, and the content ratio of polar groups to non-polar groups no longer increased, remaining at 0.51. This result further proves that the improvement of the surface activity of the composites is mainly due to the introduction of a large number of polar oxygen-containing groups on the surface of the composites. In addition, according to the bonding interface theory, the intermolecular force produced by the chemical bonding between the adhesive and bonded parts is usually stronger than that produced by mechanical interlocking. Based on the bonding interface theory and the above analysis, the interfacial bonding properties of the composites are improved after plasma treatment, mainly because the active particles activate the surface of the composites under the action of plasma. A large number of oxygen-containing active groups were introduced into the surface of the composites. The main reason for the better wettability of the composite surface after plasma treatment is also that the polar oxygen-containing functional groups are introduced into the composite surface through the plasma treatment, which significantly increases the polarity component of the composite surface and improves the surface energy.

## 4. Conclusions

(1) For the surface treatment before CF/EP bonding, whether sandblasting or the combination of sandblasting and plasma treatment, the final treatment effect is not as good as that of plasma treatment. After low-temperature plasma treatment, the tensile shear strength of a CF/EP single-lap joint increases at first and then decreases with the increase in plasma treatment time, and reaches its peak value when the treatment time is 50 s. After plasma treatment, the failure mode of the single-lap joint changes from interface failure to mixed failure dominated by cohesion failure (the combination of cohesion failure and interface failure).
(2) After low-temperature plasma surface treatment, there are clear etching marks on the surface of the CF/EP single-lap joint, which becomes rougher as a whole, mainly characterized by gully texture, pits and protruding lines and spots on the surface of carbon fiber. The increase in the bonding area of the carbon fiber and epoxy resin adhesive is beneficial for improving the mechanical interlock between the epoxy resin adhesive and carbon fiber, and makes the interface between the epoxy resin adhesive and carbon fiber tighter, which achieves the effect of improving the bonding properties of composite single-lap joints. When the plasma treatment time is too long, excessive etching will damage the carbon fiber and affect the bonding properties of the composites.
(3) After low-temperature plasma surface treatment, the wettability of the composite surface is improved. With the increase in plasma treatment time, the contact angle of the CF/EP surface decreases, the surface free energy and polarity component increase and the adsorption property between the adhesive and composite surface increase. However, due to the existence of a wetting envelope zone on the surface of the composite, when the wettability and adsorption of the zone reach saturation, the strength of the single-lap joint will not increase continuously with the increase in treatment time.
(4) After low-temperature plasma surface treatment, the O/C and N/C of the CF/EP surface increase, and a large number of polar oxygen-containing functional groups such as hydroxyl and carboxyl groups are grafted onto the surface of the composites, which improves the surface activity of the composites. With the increase in plasma treatment time, the activation degree of oxygen-containing functional groups on the surface of composites deepens, but if the treatment time is too long, a new interface layer will be formed on the surface of composites, and some of the oxygen-containing active groups will be peeled off. The surface activity of the composites no longer increases, but decreases.

## Figures and Tables

**Table 1:** Properties of EH918 resin.

Performance	Test Standard	Typical Value
Density	ASTM D792	1.26 (g/cm^3^)
Tg (DSC)	ASTM D7028	210 (°C)

**Table 2:** Properties of HF40C fiber.

Linear Density	Tensile Strength	Tensile Modulus	Elongation Rate	Density	Sizing Agent
(g/km)	(MPa)	(GPa)	(%)	(g/cm^3^)	(%)
445	5600	295	1.9	1.80	1.1

**Table 3:** Technological parameters of sandblasting.

Work Pressure (kgf/cm^2^)	Working Voltage (V)	Sand Grain Size (Mesh)	Distance (mm)	Gas Flow Rate (m^3^)
6~8	220	150	20	≥0.6

**Table 4:** Technological parameters of ion treatment.

Maximum Discharge Power (W)	Gas Source	High-Pressure Gas Flow (m^3^/min)	Nozzle Shape	Nozzle Diameter (mm)
500	Dehumidified air	6.0	Circle	8

**Table 5:** The surface energy parameters of test liquids.

Test Liquid	$\gamma_{L}^{d}\;(\mathrm{mN}/m)$	$\gamma_{L}^{p}\;(\mathrm{mN}/m)$	$\gamma_{L}\;(\mathrm{mN}/m)$
Water	21.8	51.0	72.8
Glycol	29.3	19.0	48.3

**Table 6:** Tensile shear strength of CF/EP single-lap joints with different surface treatments.

Surface Treatment Method	Average Value of Tensile Shear Strength (MPa)	Standard Deviation
Untreated	8.76	0.98
Sandblasting treatment	12.43	0.73
Plasma treatment for 25 s	16.99	0.68
Plasma treatment for 50 s	22.06	1.40
Plasma treatment for 125 s	17.08	0.94
Plasma treatment for 250 s	11.84	0.51
Sandblasting and plasma treatment for 125 s	5.71	0.63

**Table 7:** Schematic diagram of failure types of single-lap joints.

Failure Form	Interface Failure	Cohesive Failure	Substrate Failure
Schematic diagram	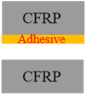	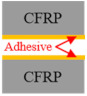	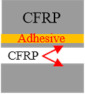

**Table 8:** Fracture morphology and failure forms of CF/EP single-lap joints with different surface treatment methods. The blue mark indicates epoxy resin glue and the white mark indicates the exposed carbon fiber substrate.

Surface Treatment Method	Fracture Morphology of Joint	Failure Form
Untreated	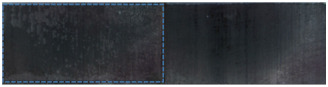	Interface failure
Sandblasting treatment	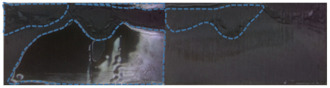	Interface failure
Plasma treatment for 25 s	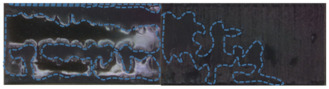	Combination of interface failure and cohesion failure
Plasma treatment for 50 s	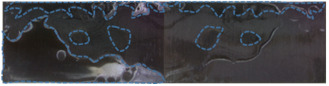	Combination of interface failure and cohesion failure
Plasma treatment for 125 s	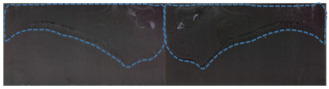	Combination of cohesion and interface failure
Plasma treatment for 250 s	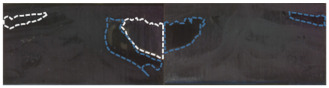	Combination of substrate failure and interface failure
Sandblasting and plasma treatment for 125 s	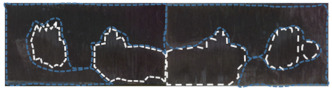	Combination of substrate failure and cohesion failure

**Table 9:** Total surface energy, polarity and dispersion components of CF/EP with different surface treatments.

Surface Treatment Method	Polar Component (mJ/m^2^)	Dispersion Component (mJ/m^2^)	Total Surface Energy (mJ/m^2^)
Untreated	3.09	20.09	23.18
Sandblasting treatment	18.86	13.71	32.57
Plasma treatment for 60 s	71.81	1.49	73.30
Plasma treatment for 120 s	77.92	0.75	78.67
Plasma treatment for 300 s	87.60	1.44	89.04
Plasma treatment for 600 s	89.99	1.17	91.16
Sandblasting and plasma treatment for 120 s	97.43	0.06	97.49

**Table 10:** Element composition and corresponding content of CF/EP sample surface after different surface treatments.

Surface Treatment Methods	Content of Elements (%)				
	C	N	O	Si	Na
Untreated	66.25	1.75	23.15	7.07	0.61
Plasma 5 s	53.57	1.82	31.63	6.89	0.48
Plasma 10 s	52.17	2.33	32.95	5.63	0.63
Plasma 25 s	49.58	3.70	33.58	6.36	0.65
Plasma 60 s	32.09	2.39	44.34	12.29	0.47
Sandblasting	60.65	1.49	26.46	4.80	0.00
Sandblasting and plasma 25 s	55.38	3.16	29.31	6.55	0.56

**Table 11:** Oxygen–carbon ratio and nitrogen–carbon ratio of CF/EP sample surface with different plasma treatment times.

Plasma Treatment Time (s)	O/C	N/C
0	0.35	0.03
5	0.59	0.03
10	0.63	0.04
25	0.68	0.07
60	1.38	0.07

**Table 12:** Types and corresponding contents of surface functional groups of CF/EP samples with different plasma treatment times.

Plasma Treatment Time (s)	Content of Functional Groups (%)			Polar/Nonpolar
	C-C	C-O/C-N	C=O/C=N	
0	78.7	14.2	7.1	0.27
5	76.9	13.9	9.2	0.30
10	71.4	19.3	9.3	0.40
25	66.2	22.5	11.3	0.51
60	66.2	24.5	9.3	0.51

## Data Availability

Not applicable.
